# Metabolic profiling of patients with different idiopathic inflammatory myopathy subtypes reveals potential biomarkers in plasma

**DOI:** 10.1007/s10238-023-01073-6

**Published:** 2023-04-27

**Authors:** Qianqian Zhao, Qiu Hu, Shuhui Meng, Qinguo Zhang, Tingting Wang, Cuilian Liu, Dongzhou Liu, Zhenyou Jiang, Xiaoping Hong

**Affiliations:** 1 Department of Rheumatology and Immunology, The Second Clinical Medical College, Jinan University (Shenzhen People’s Hospital), Shenzhen, 518020 China; 2 Integrated Chinese and Western Medicine Postdoctoral Research Station, Jinan University, Guangzhou, 510632 China; 3 The Office of Healthcare Committee of Shenzhen Municipal, Shenzhen, 518020 China; 4 Shenzhen People’s Hospital, The Frist Affiliated Hospital of Southern University of Science and Technology, Shenzhen, 518020 China; 5 Department of Microbiology and Immunology, College of Basic Medicine and Public Hygiene, Jinan University, Guangzhou, 510632 China

**Keywords:** Metabolomics, Biomarker, Idiopathic inflammatory myopathy, Anti-MDA5 positive dermatomyositis

## Abstract

Idiopathic inflammatory myopathy (IIM) are heterogeneous autoimmune diseases that primarily affect the proximal muscles. IIM subtypes include dermatomyositis (DM), polymyositis (PM), and anti-synthetase syndrome (ASS). Metabolic disturbances may cause irreversible structural damage to muscle fibers in patients with IIM. However, the metabolite profile of patients with different IIM subtypes remains elusive. To investigate metabolic alterations and identify patients with different IIM subtypes, we comprehensively profiled plasma metabolomics of 46 DM, 13 PM, 12 ASS patients, and 30 healthy controls (HCs) using UHPLC-Q Exactive HF mass spectrometer. Multiple statistical analyses and random forest were used to discover differential metabolites and potential biomarkers. We found that tryptophan metabolism, phenylalanine and tyrosine metabolism, fatty acid biosynthesis, beta-oxidation of very long chain fatty acids, alpha-linolenic acid and linoleic acid metabolism, steroidogenesis, bile acid biosynthesis, purine metabolism, and caffeine metabolism are all enriched in the DM, PM, and ASS groups. We also found that different subtypes of IIM have their unique metabolic pathways. We constructed three models (five metabolites) to identify DM, PM, ASS from HC in the discovery and validation sets. Five to seven metabolites can distinguish DM from PM, DM from ASS, and PM from ASS. A panel of seven metabolites can identify anti-melanoma differentiation-associated gene 5 positive (MDA5 +) DM with high accuracy in the discovery and validation sets. Our results provide potential biomarkers for diagnosing different subtypes of IIM and a better understanding of the underlying mechanisms of IIM.

**Supplementary Information:**

The online version contains supplementary material available at 10.1007/s10238-023-01073-6.

## Introduction

Idiopathic inflammatory myopathy (IIM) is a group of rare autoimmune disorders affecting multiple extra muscular organs in addition to the usually severe and acute muscle inflammation [1]. Despite advances in the management and treatment of IIM, they are still associated with increased morbidity and mortality, often leading to severe impairment of the quality of life [1]. The main subtypes of IIM include dermatomyositis (DM), polymyositis (PM), overlap syndrome with myositis (OM), including anti-synthetase syndrome (ASS), immune-mediated necrotizing myopathy (IMNM), and inclusion body myositis (IBM). Diagnosis and classification of different subtypes are challenging, often requiring the detection of autoantibodies, histological evaluation of skeletal muscle biopsies, and muscle MRI/EMG [2]. Improved diagnostic criteria are needed to provide a reliable diagnosis and effective treatment of all subforms of IIM. Melanoma differentiation-associated gene 5 (MDA5) is a unique autoantigen target in a subtype of DM [3]. Previous studies have found that Chinese DM patients have a higher frequency of anti-MDA5 antibodies [4]. Anti-MDA5 positive DM is generally associated with a significant risk of interstitial lung disease (ILD), which has a potentially fatal course and poor prognosis [5]. Early identification of the unique clinical condition is critical for appropriate early intervention to improve patient outcomes. Therefore, a better understanding of the pathways that trigger pathogenesis and the precise classification of IIM will aid in the clinical development of more effective treatments.

Recent studies have demonstrated that infiltration of T cells, macrophages and dendritic cells, MHC class I molecule expression on muscle fibers, and other immune mechanisms are involved in the pathophysiology of IIM [6]. Emerging evidence suggests that non-immune mechanisms such as ER stress, NFκB-activation, and free radicals also participate in skeletal muscle fiber damage in IIM [7]. However, the exact mechanism has not been elucidated. Immunometabolism is a relatively new area of metabolic research focused on functional studies between metabolic reprogramming and the immune system, which will provide an additional dimension to understanding the molecular mechanisms underlying different subtypes of IIM [8]. 18-fluorodeoxyglucose PET/CT scans reveal dysregulated glucose metabolism in the muscle of patients with DM/PM [9]. Previous studies have also shown that serum fatty acid, phosphatidylcholine, and triacylglycerol are altered in PM/DM patients compared to healthy controls [10]. Recent studies have shown that phenylalanine, tyrosine and tryptophan biosynthesis, and nitrogen metabolism are significantly involved in DM patients [11]. There is accumulating evidence that glycolysis, fatty acid oxidation, fatty acid synthesis, the tricarboxylic acid cycle, amino acid metabolism, and the pentose phosphate pathway are all involved in the metabolism of patients with IIM [12]. However, the contribution of each pathway to the pathogenesis may depend on the particular subset of IIM. Currently, information on metabolite profiles in patients with different subtypes of IIM (such as DM, PM, and ASS) and homogeneous subgroups through stratification by myositis-specific antibodies is very limited.

Here, we used an untargeted metabolomic approach to thoroughly investigate metabolite alterations in the plasma of DM, PM, and ASS patients by UHPLC-Q Exactive HF mass spectrometer. We also analyzed metabolite changes in the plasma of patients with anti-MDA5 positive (MDA5 +) and negative (MDA5-) DM. We observed differential metabolites and disturbed metabolic pathways among patients with different subtypes of IIM. Our results also show that metabolites in plasma can distinguish IIM isoforms. Our study provides new insights into the underlying mechanism of IIM, and may provide a novel diagnostic method for different subtypes of IIM.

## Materials and methods

### Materials

LC–MS-grade acetonitrile (ACN) and methanol (MeOH) were purchased from Fisher Scientific, Inc. (Rockford, IL). Formic acid was obtained from DIKMA Technologies, Inc. (Beijing, China), and ammonium formate was purchased from Sigma Aldrich (St. Louis, MO). Internal standards L-Leucine-d3 and L-Tryptophan-d5 were from Toronto Research Chemicals (Toronto, ON); L-Phenylalanine (13C9, 99%) was from Cambridge Isotope Laboratories, Inc. (Tewksbury, MA); Progesterone-2,3,4-13C3 was purchased from Sigma Aldrich (St. Louis, MO). Ultrapure water was filtered through a Milli-Q system (Millipore, Billerica, MA).

### Specimens

The samples and clinical information were reviewed and approved by the Ethics Committee of Shenzhen People’s Hospital, China (LL-KY 2019514). After obtaining informed consent, K2-EDTA plasma samples were collected from 46 patients with DM, 13 patients with PM, 12 patients with ASS, and 30 age and gender-matched healthy controls. All procedures performed in the study were in accordance with the 1964 Helsinki declaration and its later amendments. The diagnosis and classification of IIM (including DM, PM, and ASS) comply with Classification Criteria by the European League Against Rheumatism/American College of Rheumatology in 2017 [13]. At least two rheumatologists from Shenzhen People’s Hospital confirmed the diagnosis. As shown in Table 1, detailed information was collected. All plasma samples were stored at -80 °C.

**Table 1:** Demographics and baseline characteristics of patients with different IIM subtypes and healthy controls

	IIM (n = 71)			Control (n = 30)
	DM (n = 46)	PM (n = 13)	ASS (n = 12)	
Age (years) (Mean ± SD)	52 ± 13	50 ± 13	54 ± 12	50 ± 14
Gender (Male/Female)	18/28	2/11	2/10	12/18
Disease duration (weeks) (Median (IQR))	55 (20,212)	312 (52,442)	82 (52,163)	/
*Clinical characteristics (n, %)*				
Muscle weakness	33 (71.7)	13 (100.0)	9 (75.0)	/
Myalgia	14 (30.4)	9 (69.2)	4 (33.3)	/
Dysphagia	8 (17.4)	2 (15.4)	1 (8.3)	/
Arthritis	17 (37.0)	3 (23.1)	7 (58.3)	/
Periorbital edema and erythema	12 (26.1)	0 (0)	0 (0)	/
V sign	18 (39.1)	0 (0)	0 (0)	/
Shawl sign	14 (30.4)	0 (0)	0 (0)	/
Gottron papules	26 (56.5)	0 (0)	0 (0)	/
Nail-fold telangiectasias and periungual erythema	3 (6.5)	0 (0)	0 (0)	/
Mechanic's hands	13 (28.3)	0 (0)	3 (25.0)	/
Holster sign	13 (28.3)	0 (0)	0 (0)	/
Raynaud's phenomenon	2 (4.3)	0 (0)	1 (8.3)	/
Skin ulcer	6 (13.0)	0 (0)	0 (0)	/
ILD	29 (63.0)	5 (38.5)	11 (91.7)	/
*Laboratory parameters*				
ANA-positive (n, %)	21 (45.7)	6 (46.2)	10 (83.3)	/
Anti-Mi-2-positive (n, %)	6 (13.0)	0 (0)	0 (0)	/
Anti-SSA/RO52kd-positive (n, %)	21 (45.7)	5 (38.5)	9 (75.0)	/
Anti-SSB/La-positive (n, %)	2 (4.3)	1 (7.7)	0 (0)	/
Anti-RNP-positive (n, %)	3 (6.5)	2 (15.4)	1 (8.3)	/
Anti-JO-1-positive (n, %)	3 (6.5)	0 (0)	9 (75.0)	/
Anti-PL-7-positive (n, %)	2 (4.3)	0 (0)	1 (8.3)	/
Anti-PL-12-positive (n, %)	2 (4.3)	0 (0)	1 (8.3)	/
Anti-EJ-positive (n, %)	0 (0)	0 (0)	0 (0)	/
Anti-OJ-positive (n, %)	0 (0)	1 (7.7)	0 (0)	/
Anti-MDA5-positive (n, %)	17 (37.0)	0 (0)	0 (0)	/
Anti-TIF1γ-positive (n, %)	7 (15.2)	0 (0)	0 (0)	/
Anti-NXP2-positive (n, %)	2 (4.3)	0 (0)	0 (0)	/
Anti-SAE1-positive (n, %)	2 (4.3)	0 (0)	0 (0)	/
Anti-PL-Scl-positive (n, %)	1 (2.2)	0 (0)	0 (0)	/
Anti-Ku-positive (n, %)	3 (6.5)	0 (0)	0 (0)	/
Anti-SRP-positive (n, %)	4 (8.7)	2 (15.4)	0 (0)	/
Anti-HMGCR-positive (n, %)	0 (0)	0 (0)	0 (0)	/
CK (U/L) (Median (IQR))	79 (37, 281.5)	1211 (147, 1946)	105 (41, 605)	/
α-HBDH (U/L) (Median (IQR))	195 (155, 288)	312 (149, 462.5)	199 (148, 292)	/
LDH (U/L) (Median (IQR))	240 (176, 372.3)	383 (188, 556)	248 (185, 323)	/
GPT (U/L) (Median (IQR))	34 (16, 73.1)	53 (17, 94.5)	25 (13, 36.5)	/
GOT (U/L) (Median (IQR))	27 (19, 65.1)	60 (25, 88.5)	23 (15, 34)	/
CRP (mg/L) (Median (IQR))	3 (1, 6.1)	1 (0, 13)	2 (1, 10.3)	/
ESR (mm/h) (Median (IQR))	15 (8, 31.5)	9 (6, 16.5)	18 (14, 19.8)	/
ALB (g/L) (Median (IQR))	40 (34, 42.6)	39 (36, 42.8)	38 (32, 40.8)	/
ferritin (μg/L) (Median (IQR))	296 (99, 729.2)	120 (53, 275.3)	161 (71, 311.1)	/

SD, standard deviation; IQR, interquartile range; no, number; CK, creatine kinase; α-HBDH, α-hydroxybutyrate dehydrogenase; LDH, lactate dehydrogenase; GPT, glutamate pyruvic transaminase; GOT, glutamate oxaloacetate transaminase; CRP, C-reactive protein; ESR, erythrocyte sedimentation rate; ALB, serum albumin

### Metabolite extraction

The plasma metabolite extraction method followed a previous report with minor modifications [14]. Briefly, 100 µL of plasma was extracted by directly adding 300 µL of MeOH/ACN (2:1, v/v). To provide accurate quantitation of metabolites, an internal standard mix was added. Samples were vortex mixed for 1 min, then incubated at -20 °C for 2 h. Subsequently, the samples were centrifuged at 4000 rpm for 20 min, and the supernatant was transferred for vacuum freeze drying. Metabolites were resuspended with 150 µL MeOH/H_2_O (1:1, v/v) and centrifuged at 4000 rpm for 30 min. The supernatants were transferred to autosampler vials for LC–MS analysis. Equal volumes of plasma metabolite extracts were pooled as quality control (QC) samples for evaluating the performance of the entire LC–MS system.

### LC–MS/MS analysis

For the separation and detection of metabolites, a 2D UHPLC system (Waters, USA) coupled with a heated electrospray ionization (HESI) source and a Q Exactive HF mass spectrometer (Thermo Fisher Scientific, USA) was used; the LC–MS/MS system was controlled by the Xcalibur 2.3 software program. The mass data were collected in both positive and negative modes. The LC conditions were as follows: ACQUITY UHPLC BEH C18 column (2.1 × 100 mm, 1.7 μm, Waters); mobile phase, (A) 0.1% formic acid and (B) ACN (in the positive mode); (A) 10 mM ammonium formate and (B) ACN (in the negative mode). The LC gradient used was as follows: 0–1 min, 2% B; 1–9 min, 2%-98% B; 9–12 min, 98% B; 12–12.1 min, 98%-2% B; and 12.1-15 min, 2% B. The volume of injection was 5 μl. The flow rate was 0.35 ml/min, and the column temperature was maintained at 45 °C. The mass spectrometric settings for positive/negative mode were as follows: spray voltage, 3.8/-3.2 kV; sheath gas flow rate, 40; aux gas flow rate, 10; aux gas heater temperature, 350 °C; capillary temperature, 320 °C. The acquisition was performed from m/z 70 to 1050; the resolution was 70,000. The automatic gain control (AGC) target for MS acquisitions was set to 3e6. The maximum ion injection time was 100 ms. Top 3 precursors were selected for subsequent MS/MS fragmentation with a maximum ion injection time of 50 ms, a resolution of 30,000, and an AGC of 1e5. The stepped normalized collision energy was set to 20, 40, and 60 eV. To provide more reliable experimental results, the samples were randomly ordered. A QC sample was interspersed for every 10 samples (Table S1).

### Data processing

The LC–MS/MS data were preprocessed using Compound Discoverer 3.1 software, mainly including peak extraction, intra and inter-group retention time correction, missing value imputation, and compound identification. The tabular files were then processed using metaX, the metabolomics R package [15]. To obtain relative peak areas, the data were normalized by probabilistic quotient normalization (PQN). Quality control-based robust LOESS signal correction (QC-RLSC) was performed to correct the batch effect. Compounds with a coefficient of variation (CV) ≥ 30% in the QC samples were removed. All metabolites were matched against these databases, BGI self-built standard library, mzCloud, and ChemSpider (HMDB, KEGG, LipidMaps) databases. The main parameters for metabolite identification were as follows: precursor mass tolerance < 5, fragment mass tolerance < 10 ppm, and RT tolerance < 0.2 min. All identifications were further manually confirmed based on biological background. The preprocessed data were further used for the following statistical analysis.

### Statistical analysis

Multivariate and univariate statistical analyses were combined to screen for differential metabolites between groups. Principal component analysis (PCA) and partial least-squares discriminant analysis (PLS-DA) were used for sample overview and classification. To analyze similarities and differences within and between groups, as well as outliers of observed variables, PCA performed dimensionality reduction on the raw data. The PLS-DA models were validated by permutation tests (200 runs); the fitted models were considered significant when the R^2^ and Q^2^ were positive [16]. Variable importance in projection (VIP) scores were employed to visualize the effects of variables in the models. Significant differences between groups were tested using Student’s test. Differential metabolites were determined according to fold change ≥ 1.2 or ≤ 0.8, *P* value < 0.05, and VIP ≥ 1. Enrichment analysis of differential metabolites with a metabolomics standards initiative (MSI) level 1/2 was performed by MetaboAnalyst. To further discover potential biomarkers between different groups, the random forest was also used by MetaboAnalyst. When ntree was larger than 500, all the model tended to be stable; and the final random forest model parameters were set as ntree = 500 and mtry = 2.

## Results

### Idiopathic inflammatory myopathy patient characteristics and cohorts

**Fig. 1:**
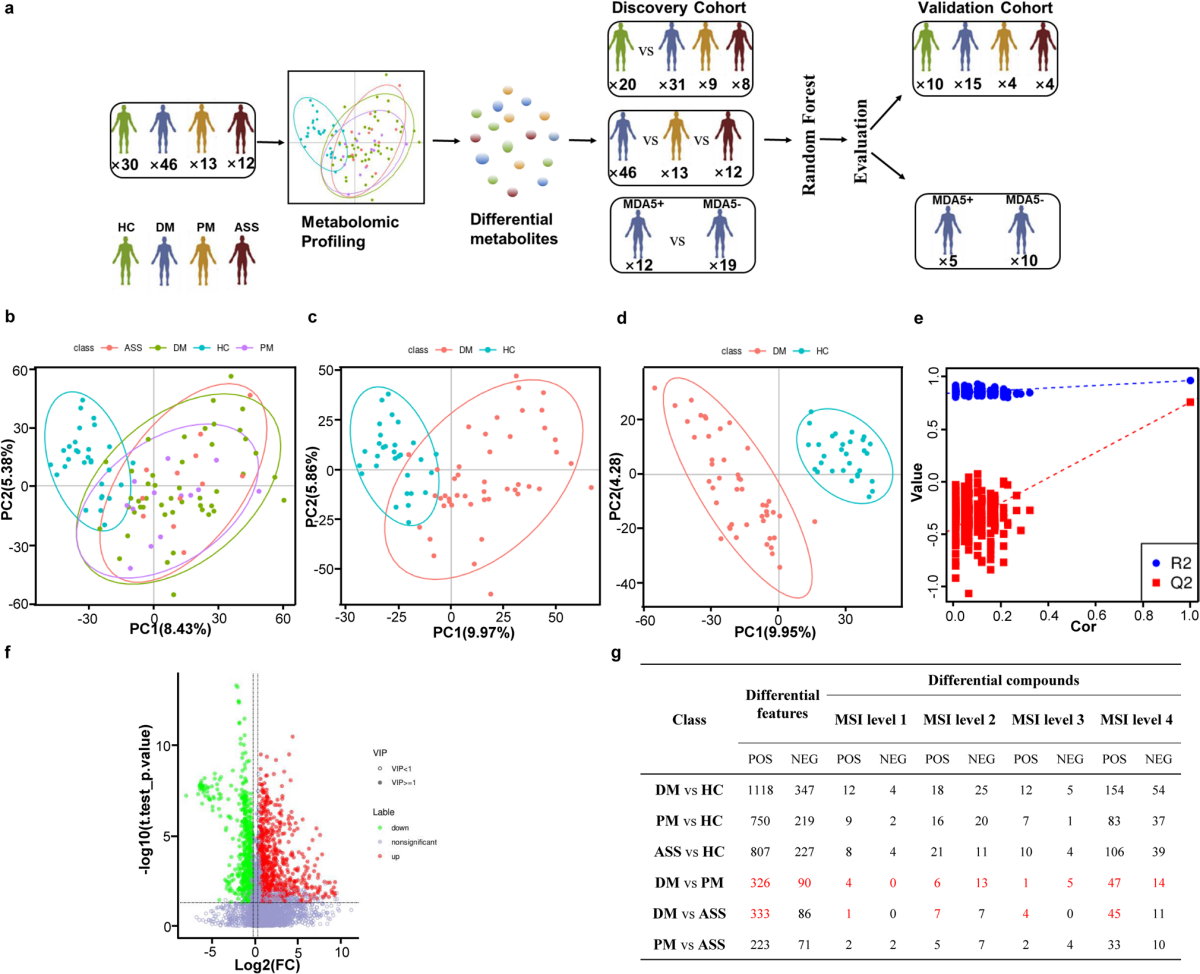
Significant differences in plasma metabolites between patients with dermatomyositis, polymyositis, anti-synthetase syndrome, and healthy controls. **a**. Idiopathic inflammatory myopathy patient cohorts. **b**. Principal component analysis (PCA) score plots from DM, PM, ASS, and HC groups. **c**. PCA score plots from DM and HC groups. **d**. Partial least square discriminant analysis (PLS-DA) score plots from DM and HC groups. **e**. PLS-DA models were evaluated by 200 permutation tests. **f**. Volcano plots from DM and HC groups. **g**. Summary of differential features and metabolites between patients with different IIM subtypes and HCs. POS, positive; NEG, negative

To evaluate changes of metabolites in the plasma of DM, PM, ASS patients, and HCs, we recruited 46, 13, and 12 patients with DM, PM, and ASS, and 30 HCs, respectively. Detailed demographics, clinical characteristics, and laboratory parameters of patients with IIM and HC are shown in Table 1. Thirty-seven percent of DM patients were anti-MDA5 positive. To find biomarkers for the diagnosis of DM, PM, ASS patients from HCs, we divided the samples into a discovery (DM, n = 31; PM, n = 9; ASS, n = 8; HC, n = 20) and a validation (DM, n = 15; PM, n = 4; ASS, n = 4; HC, n = 10) cohort. In order to discover metabolite biomarkers for predicting MDA5 + DM patients, we also divided DM patients into discovery (MDA5 + , n = 12; MDA5-, n = 19) and validation (MDA5 + , n = 5; MDA5-, n = 10) groups based on MDA5 positive or negative (Fig. 1a). Moreover, the different groups used in this study are properly matched for age and gender in both the discovery and validation sets (Table S2). All the samples were analyzed in the same batch.

### Significant differences in plasma metabolites between dermatomyositis, polymyositis, anti-synthetase syndrome patients and healthy controls

To investigate differential metabolites in different subtypes of IIM patients and HCs, we systematically analyzed metabolites in plasma from DM, PM, ASS patients, and HCs by untargeted metabolomics using high-resolution LC–MS. The quality of metabolite extraction and LC–MS analysis was assessed by base peak chromatogram (BPC) overlay of QC samples, PCA of all samples, CVs of features (retention time (RT) and m/z) in QC samples and internal standards. The BPCs of QC samples overlapped well, and QC samples were tightly clustered in the PCA plots of all samples (Fig.S1 and S2). The percentages of features with CV < 30% detected in positive and negative modes were 88.02% and 88.24%, respectively (Fig.S3). The CVs of all internal standards were less than 20%. The results showed that the data quality is sufficient for the following metabolomics profiling.

We first established PCA to observe the distribution of features in DM, PM, ASS, and HC groups. The PCA plot showed that the IIM groups (including DM, PM, and ASS) were well separated from the HC groups, and no abnormal samples were found (Fig. 1b). PCA loading plot was shown in Fig.S4a.We further applied PCA and PLS-DA to reveal discriminative features between the two groups, including DM vs. HC, PM vs. HC, ASS vs. HC, DM vs. PM, DM vs. ASS, and PM vs. ASS. The PCA plots with features were well separated between DM, PM, ASS, and HC groups (Fig. 1c, S5a1, and S5a2), whereas different subtypes of IIM couldn’t be separated (Fig.S5a3-a5). PCA loading plots were shown in Fig.S4b and Fig.S5b1-b5. PLS-DA plots showed that the two groups (including DM vs. HC, PM vs. HC, ASS vs. HC, DM vs. PM, DM vs. ASS, and PM vs. ASS) were well separated; and PLS-DA models were evaluated by 200 permutation tests (Fig. 1d, e, S5c1-c5). These models were robust in DM vs. HC, PM vs. HC, ASS vs. HC, and PM vs. ASS groups (DM vs. HC: R^2^, 0.96; Q^2^, 0.76; PM vs. HC: R^2^, 0.98; Q^2^, 0.73; ASS vs. HC: R^2^, 0.99; Q^2^, 0.76; PM vs. ASS: R^2^, 1; Q^2^, 0.1;). However, these models overfitted in DM vs. PM (R^2^, 0.92; Q^2^, − 0.22;) and DM vs. ASS groups (R^2^, 0.94; Q^2^, − 0.33). These VIP values in these overfitted models were not adopted in the following analysis.

We used the following criteria: fold change ≥ 1.2 or ≤ 0.8, *P* value < 0.05, and VIP ≥ 1 to find differential features in different subtypes of IIM patients and HCs. Differential features were visualized by volcano plots (Fig. 1f, S5d1-d5). As shown in Fig. 1g, we found 1465, 969, 1034, 416, 419, and 294 features with significant differences in the DM vs. HC, PM vs. HC, ASS vs. HC, DM vs. PM, DM vs. ASS, and PM vs. ASS groups, respectively (We adopted fold change ≥ 1.2 or ≤ 0.8, and *P* value < 0.05 to find differential features in DM vs. PM, and DM vs. ASS groups). After identifying these features, 59, 47, 44, 23, 15, and 16 differential metabolites with MSI level 1/2 were identified in the DM vs. HC, PM vs. HC, ASS vs. HC, DM vs. PM, DM vs. ASS, and PM vs. ASS groups, respectively (Fig. 1g). All results indicated that metabolites in plasma between DM, PM, ASS, and HC are significantly different, contributing to a comprehensive understanding of the role of metabolites in the pathogenesis of different subtypes of IIM.

### Three models with five metabolites can identify dermatomyositis, polymyositis, and anti-synthetase syndrome from healthy controls

**Fig. 2:**
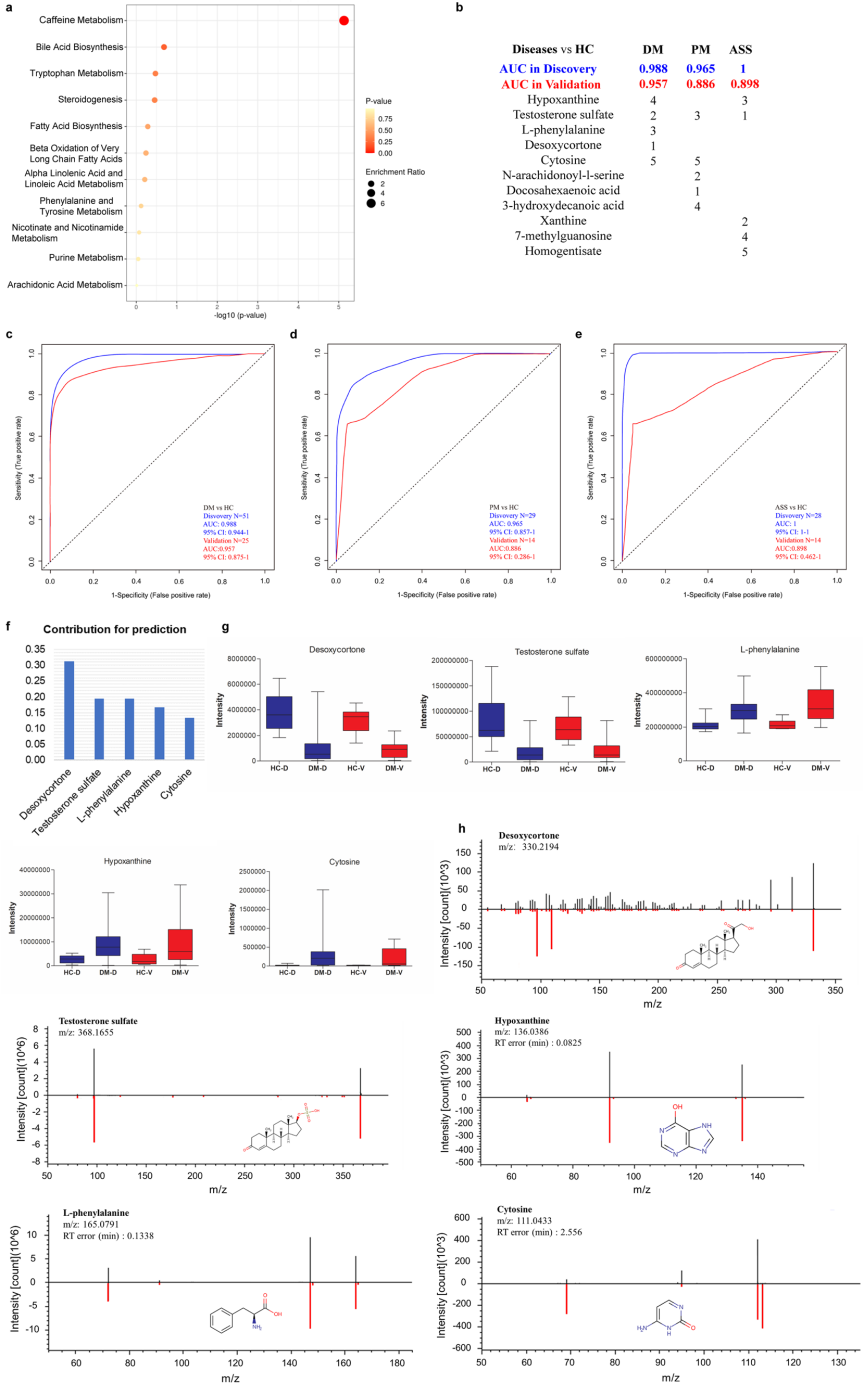
Five metabolites selected by random forest can identify dermatomyositis, polymyositis, and anti-synthetase syndrome from healthy controls. **a**. Enrichment analysis of differential metabolites with MSI level 1/2 between DM and HC groups. **b**. Summary of models of identifying DM, PM, and ASS from HC using five metabolites in plasma in the discovery and validation cohorts. The contribution rank of each metabolite in every model is listed as number 1, 2, …, and 5. The receiver operating characteristic (ROC) curve based on five metabolites can accurately identify DM (**c**), PM (**d**), and ASS (**e**) from HC in the discovery and validation sets. **f**. Contribution of five metabolites to the identification model of DM. g. The concentration trends of individual metabolites in the DM and HC groups in the discovery and validation sets. **h**. Detailed MS/MS spectra of five potential metabolite biomarkers for identification of DM. The measured MS/MS spectral fragment profile (top, black) matching the commercial standard/theoretical fragment (bottom, red); HC-D, DM-D, HC and DM groups in the discovery set, respectively; HC-V, DM-V, HC and DM groups in the validation set, respectively

To detect pathway changes in different subtypes of IIM, enrichment analysis was performed using differential metabolites with MSI level 1/2 in the DM vs. HC, PM vs. HC, and ASS vs. HC groups. As shown in Fig. 2a, S6a1, and S6a2, multiple biological processes were enriched in different subtypes of IIM patients; caffeine metabolism, bile acid biosynthesis, tryptophan metabolism, steroidogenesis, fatty acid biosynthesis, beta-oxidation of very long chain fatty acids, alpha-linolenic acid and linoleic acid metabolism, phenylalanine and tyrosine metabolism, and purine metabolism were all enriched in the DM, PM, and ASS groups. To assess the discriminative ability of metabolites to diagnose patients with DM, PM, and ASS from HCs, we applied feature selection with differential metabolites with MSI level 1/2 to construct models in the discovery set (DM, n = 31; PM, n = 9; ASS, n = 8; HC, n = 20) by random forest. We then tested the independent performance of these models in the validation set (DM, n = 15; PM, n = 4; ASS, n = 4; HC, n = 10). As shown in Fig. 2b and c, the panel of five metabolites (Desoxycortone, Testosterone sulfate, L-phenylalanine, Hypoxanthine, and Cytosine) could accurately identify DM patients from HCs in both the discovery (AUC = 0.988) and validation (AUC = 0.957) sets. The contributions of these five metabolites to the model are shown in Fig. 2f. We further analyzed the concentration trends of these metabolites in the DM and HC groups in both the discovery and validation sets, respectively. As shown in Fig. 2g, the changes in metabolite concentrations between the DM and HC groups in the discovery and validation sets were consistent. Similarly, five metabolites (PM: Docosahexaenoic acid, N-arachidonoyl-l-serine, Testosterone sulfate, 3-hydroxydecanoic acid, and Cytosine; ASS: Testosterone sulfate, Xanthine, Hypoxanthine, 7-methylguanosine, and Homogentisate) could identify patients with PM, and ASS from HCs in both the discovery and validation cohorts (Fig. 2b, d, e). Interestingly, the metabolites partially overlapped between these models, while the contributions to the models and the concentration trends of individual metabolites differed (Fig.S6b1-b2, c1-c2). The metabolite identifications were confirmed by commercial standards; if commercial standards were lacking, metabolites were further confirmed manually by spectral matching to the theoretical fragments (Fig. 2h, S6d1-d2). Together, all results suggested that we can accurately identify patients with DM, PM, and ASS from HCs by using metabolites in plasma.

### Five to seven metabolites in plasma can distinguish between different subtypes of idiopathic inflammatory myopathy

Next, we applied enrichment analysis to examine pathway changes in the DM vs. PM, DM vs. ASS, and PM vs. ASS groups, using differential metabolites with MSI level 1/2.

**Fig. 3:**
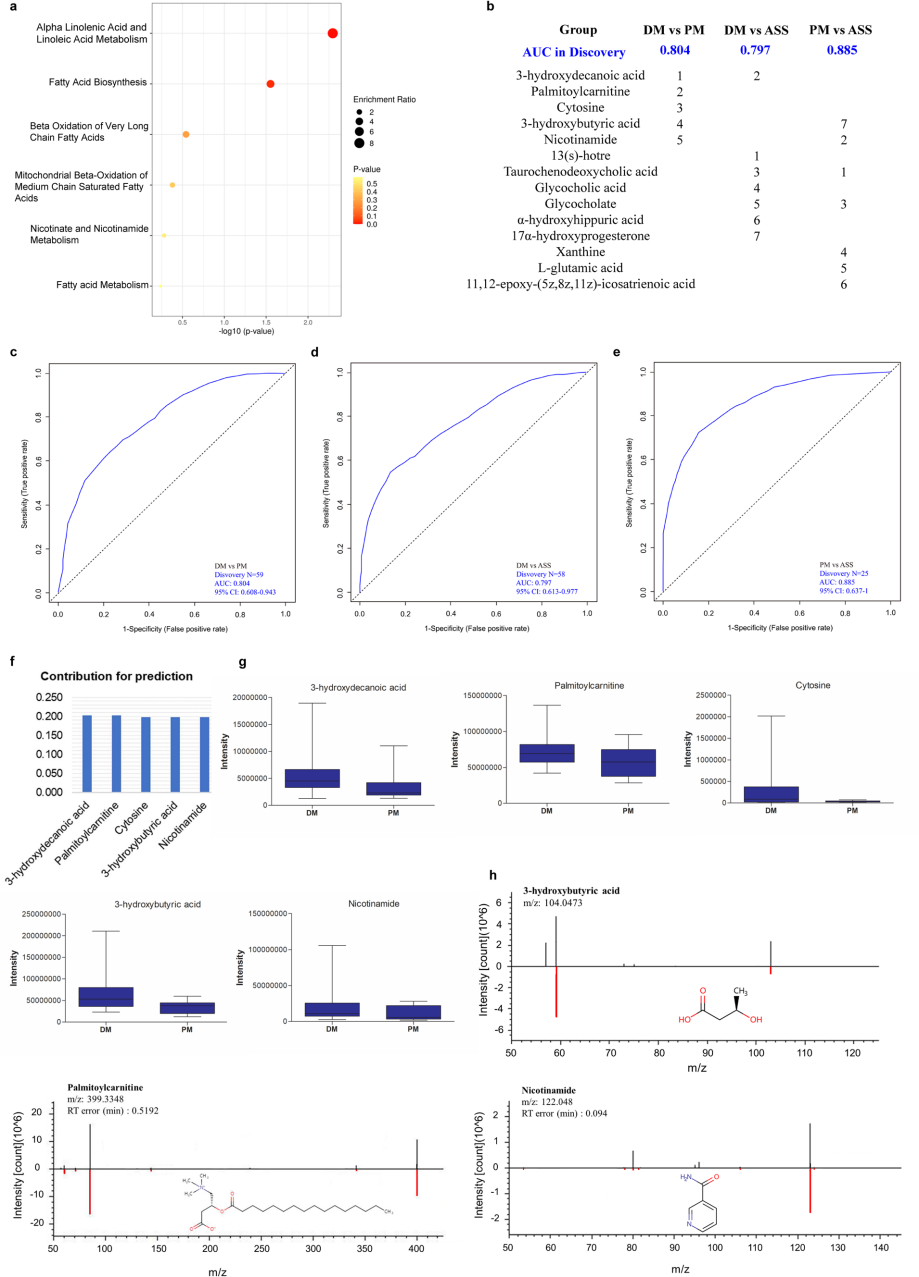
Five to seven metabolites selected by random forest can distinguish different idiopathic inflammatory myopathy subtypes. **a**. Enrichment analysis of differential metabolites with MSI level 1/2 between DM and PM groups. **b**. Summary of models of differentiating DM, PM, and ASS using five to seven metabolites in plasma. The contribution rank of each metabolite in every model is listed as number 1, 2, …, and 7. ROC curves based on five to seven metabolites can accurately distinguish DM from PM (**c**), DM from ASS (**d**), and PM from ASS (**e**). **f**. Contribution of five metabolites to the identification model of DM from PM. **g**. The concentration trends of individual metabolites in the DM and PM groups. **h**. Detailed MS/MS spectra of 3-hydroxydecanoic acid, Palmitoylcarnitine, and Nicotinamide

As shown in Fig. 3a, S7a1, and S7a2, different metabolic pathways were enriched in different subtypes of IIM patients; however, fatty acid biosynthesis was enriched in all cases. We also evaluated whether metabolites in plasma could distinguish between different subtypes of IIM patients in the discovery set (DM, n = 46; PM, n = 13; ASS, n = 12) using differential metabolites with MSI level 1/2 by random forest. As shown in Fig. 3b and c, a panel of five metabolites (3-hydroxydecanoic acid, Palmitoylcarnitine, Cytosine, 3-hydroxybutyric acid, and Nicotinamide) has an AUC of 0.804 to differentiate DM from PM. The predicted contributions of these five metabolites are shown in Fig. 3f. The concentrations of these five metabolites in the DM and PM groups are shown in Fig. 3g. Detailed MS/MS spectra of 3-hydroxydecanoic acid, Palmitoylcarnitine, Cytosine, and Nicotinamide are shown in Fig. 3h. We also examined metabolites in plasma to differentiate DM from ASS, and PM from ASS. We found that seven metabolites in plasma (DM vs. ASS: 13(s)-hotre, 3-hydroxydecanoic acid, Taurochenodeoxycholic acid, Glycocholic acid, Glycocholate, α-hydroxyhippuric acid, and 17α-hydroxyprogesterone; PM vs. ASS: Taurochenodeoxycholic acid, Nicotinamide, Glycocholate, Xanthine, L-glutamic acid, 11,12-epoxy-(5z,8z,11z)-icosatrienoic acid, and 3-hydroxybutyric acid) could distinguish these two groups (DM vs. ASS: AUC = 0.797; PM vs. ASS: AUC = 0.885) (Fig. 3b, d, and e). Similar to the above results, the metabolites partially overlapped, and the contributions and concentrations of individual metabolites varied (Fig. 3b, S7b1-b2, c1-c2). Detailed MS/MS spectra of 13(s)-hotre, Taurochenodeoxycholic acid, Glycocholic acid, Glycocholate, α-hydroxyhippuric acid, 17α-hydroxyprogesterone, L-glutamic acid, and 11,12-epoxy-(5z,8z,11z)-icosatrienoic acid are shown in Fig.S7d1-d2. These results demonstrated that metabolites in plasma contain information on distinguishing between different subtypes of IIM patients.

### A panel of seven metabolites can predict MDA5 + dermatomyositis

**Fig. 4:**
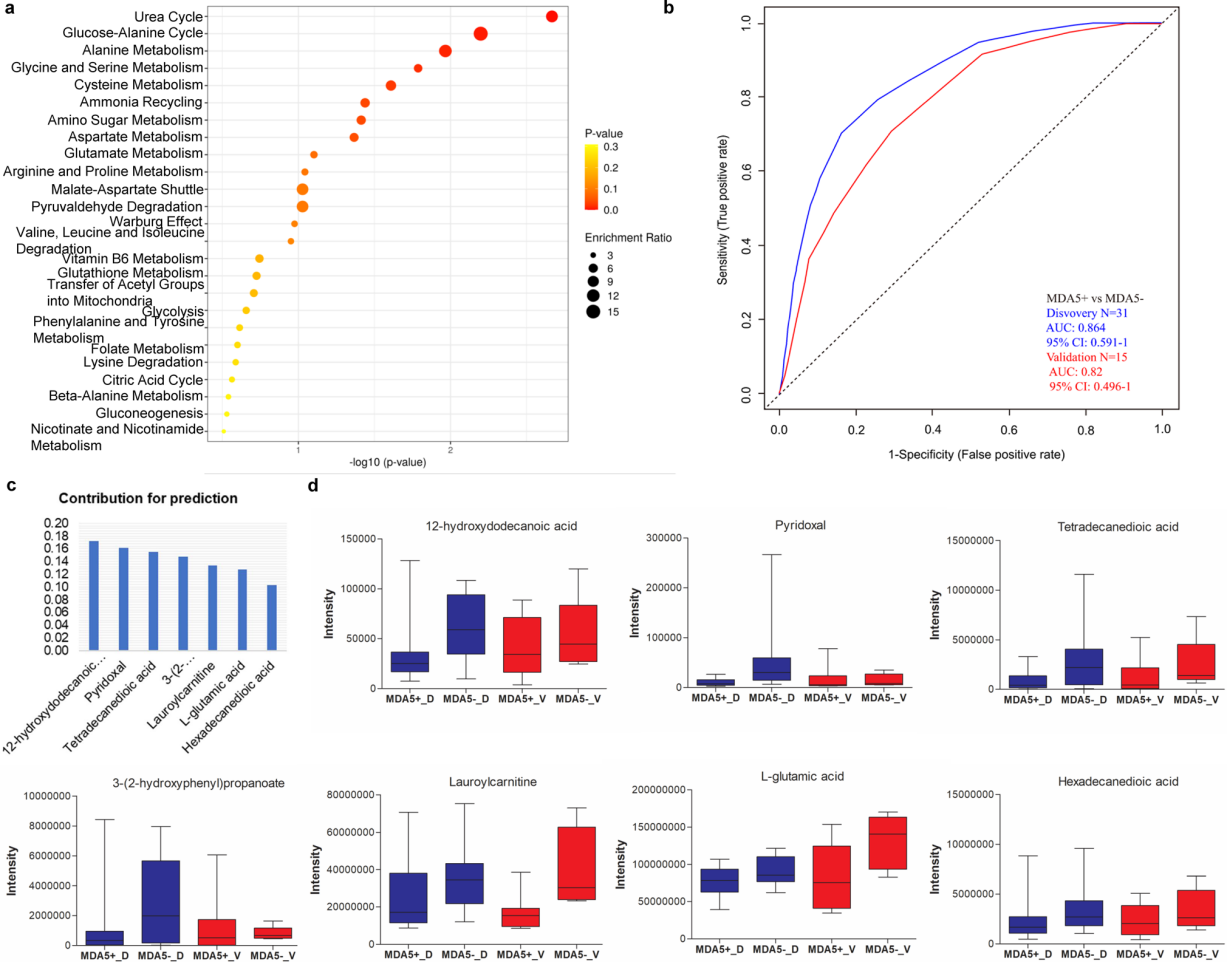
Seven metabolites selected by random forest can predict MDA5 + dermatomyositis. **a**. Enrichment analysis of differential metabolites with MSI level 1/2 between MDA5 + and MDA5-DM. **b**. ROC curve based on seven metabolites can accurately predict MDA5 + DM. **c**. Contribution of seven metabolites to the prediction model of MDA5 + DM. **d**. The concentration trends of individual metabolites in the MDA5 + and MDA5-DM groups in the discovery and validation sets. MDA5 + _D, MDA5-_D, MDA5 + and MDA5-DM groups in the discovery set, respectively; MDA5 + _V, MDA5-_V, MDA5 + and MDA5-DM groups in the validation set, respectively

We next investigated metabolite alterations in the plasma of MDA5 + and MDA5- DM patients. We found that 342 features were significantly different between MDA5 + and MDA5- DM patients. Ten metabolites with MSI level 1/2 were identified. The pathways involved in these metabolites are shown in Fig. 4a. To evaluate the ability of metabolites in plasma to discriminate between patients with MDA5 + and MDA5- DM, we established models with these ten metabolites in the discovery cohort (MDA5 + , n = 12; MDA5-, n = 19) by random forest and tested the model in the validation cohort (MDA5 + , n = 5; MDA5-, n = 10). As shown in Fig. 4b, a panel of seven metabolites (12-hydroxydodecanoic acid, Pyridoxal, Tetradecanedioic acid, 3-(2-hydroxyphenyl)propanoate, Lauroylcarnitine, L-glutamic acid, and Hexadecanedioic acid) could precisely distinguish MDA5 + DM patients from MDA5- DM patients in both the discovery (AUC = 0.864) and validation (AUC = 0.82) cohorts. The contributions of these seven metabolites are shown in Fig. 4c. The metabolite concentration alternatives were consistent between the MDA5 + and MDA5- DM groups in the discovery and validation sets (Fig. 4d). Detailed MS/MS spectra of these seven metabolites are shown in Fig.S8. These results suggested that the metabolism of MDA5 + DM patients differs from that of MDA5- DM patients, and the metabolites in plasma are predictive of MDA5 + DM patients.

## Discussion

Here, we performed untargeted metabolomics analysis to identify metabolite changes in different IIM subtypes. Since anti-MDA5 antibodies showed great utility in identifying DM patients with higher homogeneity, we also analyzed metabolites associated with MDA5 + DM patients. We further estimated the power of five to seven candidate metabolite biomarkers to differentiate DM, PM, and ASS patients from HCs, between different subtypes of IIM patients, and MDA5 + and MDA5- DM patients in the discovery/validation cohorts by random forest.

These metabolite alterations presumably reflect essential biological physiology of different IIM subgroups. Here, we found that tryptophan metabolism, phenylalanine and tyrosine metabolism, fatty acid biosynthesis, beta-oxidation of very long chain fatty acids, alpha-linolenic acid and linoleic acid metabolism, steroidogenesis, bile acid biosynthesis, purine metabolism, and caffeine metabolism are all enriched in the DM, PM, and ASS groups. Consistent with these results, Tie Zhang et al. found that phenylalanine, tyrosine and tryptophan biosynthesis are the most prominently altered pathways in DM [11]. Oxidative metabolism of tryptophan increases when inflammation occurs [17]. Previous studies have shown that tyrosine, phenylalanine, and tryptophan are also associated with several cancers [18]. Tyrosine is formed from phenylalanine catalyzed by phenylalanine hydroxylase. Neurauter, G. et al. found that phenylalanine hydroxylase activity is changed in malignancy [19]. In agreement with our results, Joan Raouf et al. found that serum lipids profiles are significantly altered in DM/PM patients [10]. There is increasing evidence that various lipids, such as fatty acids, phospholipids, and prostaglandins, play important roles in regulating skeletal muscle growth and functions [20, 21]. Altered lipid metabolism may be caused by chronic inflammation and ER stress. Lipidomic disturbances lead to persistent muscle impairment [22, 23]. Previous studies have suggested that peroxidation of plasma membrane lipids may account for the mechanism of abundant production of reactive oxygen intermediates in IIM [24]. Purine metabolism has also been implicated in the physiopathology of IIM. Recent studies have shown that purine nucleoside phosphorylase (PNP) deficiency is associated with an increased risk of developing autoimmune disorders, such as lupus [25] and autoimmune hemolytic anemia [26]. Additionally, high levels of cytosine can be found in the urine of individuals with severe combined immunodeficiency syndrome [27]. Caffeine can interact with different components of the immune system by acting as a non-specific phosphodiesterase inhibitor [28]. It affects the immune system by inhibiting the release of proinflammatory cytokines, influencing the activity of macrophages and natural killer cells, reducing the proliferation of T and B cells proliferation and ultimately producing antibodies [29]. Caffeine metabolism is associated with disease activity, severity, and constitutional symptoms of autoimmune diseases, such as systemic lupus erythematosus [30] and rheumatoid arthritis [31]. We also found that arachidonic acid metabolism is enriched in DM and ASS patients. Arachidonic acid metabolites, such as leukotriene subfamilies and prostaglandin, contribute to muscular pain and inflammation, myogenesis, and muscle repair in chronic inflammatory diseases [32]. More importantly, we also found that different subtypes of IIM have their unique metabolic pathways, suggesting that metabolites in plasma can define IIM subtypes. However, further studies will be required to explore the detailed metabolite mechanisms associated with different IIM subtypes.

Here, we established three models with five metabolites in plasma that can identify DM, PM, and ASS patients from HCs. Notably, we split the samples into discovery and validation cohorts to test the independent performance of these models. We found that the panels of five metabolites are sufficient to distinguish DM, PM, and ASS patients from HCs in the discovery and validation sets. To ensure the accuracy of identification of these metabolites, we confirmed by commercial standards or theoretical fragments. MS/MS spectra of these metabolites showed that the identification is reliable. Despite the small sample size of the PM and ASS groups, we also assessed the ability of the metabolites in plasma to discriminate between different IIM subgroups. In this study, we found that five to seven metabolites in plasma can discriminate between different subtypes of IIM patients with high accuracy in the discovery cohort. Taken together, the metabolites in plasma contain information to distinguish different IIM subtypes. However, these potential metabolic biomarkers require further validation in independent and larger cohorts.

In this study, we found that metabolites in plasma are significantly different between MDA5 + and MDA5- DM patients. Our results also suggested that a panel of seven metabolites can accurately predict MDA5 + DM patients in both the discovery and validation cohorts. The subset of DM with MDA5 + antibodies present considerable challenges to rheumatologists in the clinical management of DM patients [33]. Anti-MDA5 antibodies are demonstrated to be closely linked to ILD with poor survival [34]. Previous studies have shown that activation of monocytes, macrophages [35], neutrophils, and CD4 + T helper (Th)1 cells, as well as increased expression of CD4 + CXCR4 + T cells [36] and interferon (IFN)-g [37] contribute to the development of DM-ILD. Our study provides a new perspective for understanding the non-immune molecular mechanisms of MDA5 + DM [38].

## Conclusions

In conclusion, our findings provide evidence that metabolic homeostasis is disrupted in IIM patients compared with age and gender-matched HCs; in addition, combinatorial analysis of five to seven metabolites in plasma possibly allows for distinguishing DM, PM, and ASS patients from HCs, between different subtypes of IIM patients, and even MDA5 + and MDA5- DM patients. Our insights into plasma metabolite differences between different IIM subtypes highlight the importance of metabolite variation in the pathogenesis of IIM. Future studies will elucidate whether changes in plasma metabolomic profiles reflect muscle tissue metabolite profiles and validate whether these metabolites can be used as diagnostic biomarkers for clinical IIM subtypes in larger cohorts.

### Supplementary Information

Below is the link to the electronic supplementary material.

Supplementary file1 (DOCX 4597 KB)

## References

[1] Dalakas MC (2015). Inflammatory muscle diseases. N Engl J Med.

[2] Schmidt J (2018). Current classification and management of inflammatory myopathies. J Neuromuscul Dis..

[3] Sato S, Hirakata M, Kuwana M (2005). Autoantibodies to a 140-kd polypeptide, CADM-140, in Japanese patients with clinically amyopathic dermatomyositis. Arthritis Rheum.

[4] Chen Z, Hu W, Wang Y (2015). Distinct profiles of myositis-specific autoantibodies in Chinese and Japanese patients with polymyositis/dermatomyositis. Clin Rheumatol.

[5] Abe Y, Matsushita M, Tada K (2017). Clinical characteristics and change in the antibody titres of patients with anti-MDA5 antibody-positive inflammatory myositis. Rheumatology (Oxford).

[6] Herbelet S, De Bleecker JL (2018). Immune checkpoint failures in inflammatory myopathies: An overview. Autoimmun Rev.

[7] Manole E, Bastian AE, Butoianu N, Goebel HH (2017). Myositis non-inflammatory mechanisms: An up-dated review. J Immunoassay Immunochem.

[8] Takeshima Y, Iwasaki Y, Fujio K, Yamamoto K (2019). Metabolism as a key regulator in the pathogenesis of systemic lupus erythematosus. Semin Arthritis Rheum.

[9] Li Y, Zhou Y, Wang Q (2017). Multiple values of (18)F-FDG PET/CT in idiopathic inflammatory myopathy. Clin Rheum.

[10] Raouf J, Idborg H, Englund P (2018). Targeted lipidomics analysis identified altered serum lipid profiles in patients with polymyositis and dermatomyositis. Arthritis Res Ther.

[11] Zhang T, Xu J, Liu Y, Liu J (2019). Metabolomic profiling for identification of potential biomarkers in patients with dermatomyositis. Metabolomics.

[12] Marie I (2012). Morbidity and mortality in adult polymyositis and dermatomyositis. Curr Rheumatol Rep.

[13] Lundberg IE, Tjärnlund A, Bottai M (2017). 2017 european league against rheumatism/american college of rheumatology classification criteria for adult and juvenile idiopathic inflammatory myopathies and their major subgroups. Ann Rheum Dis.

[14] Dunn WB, Broadhurst D, Begley P (2011). Procedures for large-scale metabolic profiling of serum and plasma using gas chromatography and liquid chromatography coupled to mass spectrometry. Nat Protoc.

[15] Wen B, Mei Z, Zeng C, Liu S (2017). metaX: a flexible and comprehensive software for processing metabolomics data. BMC Bioinform.

[16] Zhao Q, Ma Z, Wang X (2020). Lipidomic Biomarkers of Extracellular Vesicles for the Prediction of Preterm Birth in the Early Second Trimester. J Proteom Res..

[17] Palego L, Betti L, Rossi A, Giannaccini G (2016). Tryptophan biochemistry: structural, nutritional, metabolic, and medical aspects in humans. J Amino Acids.

[18] Lai HS, Lee JC, Lee PH, Wang ST, Chen WJ (2005). Plasma free amino acid profile in cancer patients. Semin Cancer Biol.

[19] Neurauter G, Grahmann AV, Klieber M (2008). Serum phenylalanine concentrations in patients with ovarian carcinoma correlate with concentrations of immune activation markers and of isoprostane-8. Cancer Lett.

[20] Lipina C, Hundal HS (2017). Lipid modulation of skeletal muscle mass and function. J Cachexia Sarcopenia Muscle.

[21] Funai K, Lodhi IJ, Spears LD (2016). Skeletal muscle phospholipid metabolism regulates insulin sensitivity and contractile function. Diabetes.

[22] Han J, Kaufman RJ (2016). The role of ER stress in lipid metabolism and lipotoxicity. J Lipid Res.

[23] Loell I, Raouf J, Chen YW (2016). Effects on muscle tissue remodeling and lipid metabolism in muscle tissue from adult patients with polymyositis or dermatomyositis treated with immunosuppressive agents. Arthritis Res Ther.

[24] Stangel M, Mix E, Zettl UK, Gold R (2001). Oxides and apoptosis in inflammatory myopathies. Microsc Res Tech.

[25] Ghodke-Puranik Y, Dorschner JM, Vsetecka DM (2017). Lupus-associated functional polymorphism in pnp causes cell cycle abnormalities and interferon pathway activation in human immune cells. Arthritis Rheumatol.

[26] Rich KC, Arnold WJ, Palella T, Fox IH (1979). Cellular immune deficiency with autoimmune hemolytic anemia in purine nucleoside phosphorylase deficiency. Am J Med.

[27] Mills GC, Schmalstieg FC, Newkirk KE, Goldblum RM (1979). Cytosine and orotic acid in urine of immunodeficient children. Clin Chem.

[28] Aronsen L, Orvoll E, Lysaa R, Ravna AW, Sager G (2014). Modulation of high affinity ATP-dependent cyclic nucleotide transporters by specific and non-specific cyclic nucleotide phosphodiesterase inhibitors. Eur J Pharmacol.

[29] Sharif K, Watad A, Bragazzi NL (2017). Coffee and autoimmunity: more than a mere hot beverage!. Autoimmun Rev.

[30] Orefice V, Ceccarelli F (2020). Caffeine intake influences disease activity and clinical phenotype in systemic lupus erythematosus patients. Lupus.

[31] Ingegnoli F, Cavalli S, Giudice L, Caporali R (2022). Caffeine and rheumatoid arthritis: A complicated relationship. Autoimmun Rev.

[32] Korotkova M, Lundberg IE (2014). The skeletal muscle arachidonic acid cascade in health and inflammatory disease. Nat Rev Rheumatol.

[33] Chen F, Wang D, Shu X, Nakashima R, Wang G (2012). Anti-MDA5 antibody is associated with A/SIP and decreased T cells in peripheral blood and predicts poor prognosis of ILD in Chinese patients with dermatomyositis. Rheumatol Int.

[34] Johnson C, Pinal-Fernandez I, Parikh R (2016). Assessment of mortality in autoimmune myositis with and without associated interstitial lung disease. Lung.

[35] Zuo Y, Ye L, Liu M (2020). Clinical significance of radiological patterns of HRCT and their association with macrophage activation in dermatomyositis. Rheumatology (Oxford).

[36] Wang K, Zhao J, Chen Z (2019). CD4+CXCR4+ T cells as a novel prognostic biomarker in patients with idiopathic inflammatory myopathy-associated interstitial lung disease. Rheumatology (Oxford).

[37] Ishikawa Y, Iwata S, Hanami K (2018). Relevance of interferon-gamma in pathogenesis of life-threatening rapidly progressive interstitial lung disease in patients with dermatomyositis. Arthritis Res Ther.

[38] Zhu H, Chen W, Liu D, Luo H (2019). The role of metabolism in the pathogenesis of systemic sclerosis. Metabolism.

