# Supplementary material for: Metabolic profiling of patients with different idiopathic inflammatory myopathy subtypes reveals potential biomarkers in plasma
Source: Clin Exp Med. 2023 Apr 27;23(7):3417–29. doi: 10.1007/s10238-023-01073-6 (PMC10618316; doi:10.1007/s10238-023-01073-6)
Supplement: Supplementary file 1 — Supplementary file1 (DOCX 4597 KB) [file 10238_2023_1073_MOESM1_ESM.docx]

**Supporting Information_** **Metabolic profiling of patients with different idiopathic inflammatory myopathy subtypes reveals potential biomarkers in plasma**

Qianqian Zhao^1,2,†^, Qiu Hu^1,†^, Shuhui Meng^1^, Qinguo Zhang^3^,Tingting Wang^1,2^, Cuilian Liu^1^, Dongzhou Liu^1,2,4^, Zhenyou Jiang^5*^, and Xiaoping Hong^1,2,4*^

^1^ Department of Rheumatology and Immunology, The Second Clinical Medical College, Jinan University (Shenzhen People's Hospital), Shenzhen 518020, China.

^2^ Integrated Chinese and Western Medicine Postdoctoral Research Station, Jinan University, Guangzhou 510632, China.

^3^ The office of Healthcare Committee of Shenzhen Municipal, Shenzhen 518020, China.

^4^ Shenzhen People's Hospital, The Frist Affiliated Hospital of Southern University of Science and Technology, Shenzhen, 518020, China.

^5^ Department of Microbiology and Immunology, College of Basic Medicine and Public Hygiene, Jinan University, Guangzhou, 510632, China.

^†^ These authors contributed equally to this work.

**^*^** Correspondence: Xiaoping Hong, Department of Rheumatology and Immunology, The Second Clinical Medical College, Jinan University (Shenzhen People's Hospital), Shenzhen 518020, China; Integrated Chinese and Western Medicine Postdoctoral Research Station, Jinan University, Guangzhou 510632, China; Shenzhen People's Hospital, The Frist Affiliated Hospital of Southern University of Science and Technology, Shenzhen, 518020, China. E-mail: hong_xiaoping@hotmail.com.

Zhenyou Jiang, Department of Microbiology and Immunology, College of Basic Medicine and Public Hygiene, Jinan University, Guangzhou, 510632, China. E-mail: tjzhy@jnu.edu.cn.

**Table of Content**

Fig.S1……………………………………………………………………………………………….... S-2

Fig.S2………………………………………………………………………………………………… S-2

Fig.S3………………………………………………………………………………………………… S-2

Fig.S4……………………………………………………………………………………………….... S-3

Fig.S5…………………………………………………………………………….……………………S-4

Fig.S6…………………………………………………………………………….……………………S-6

Fig.S7…………………………………………………………………………….……………………S-8

Fig.S8…………………………………………………………………………….……………………S-9

Table S1 …………………………………………………………………………….………………. S-10

Table S2 …………………………………………………………………………….………………. S-13


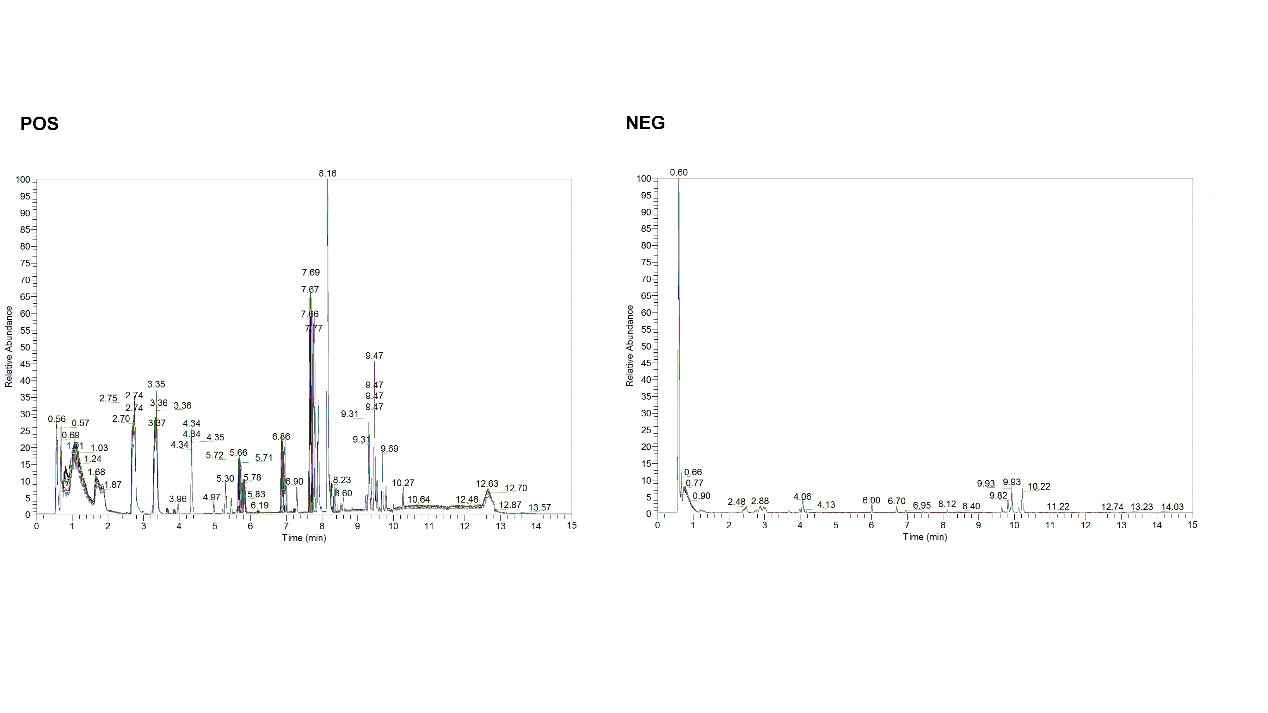


**NEG**

**POS**

**Fig.S1 Base peak chromatogram (BPC) of QC samples (pooled metabolite extracts of plasma) in the positive and negative modes.**

**NEG**

**POS**


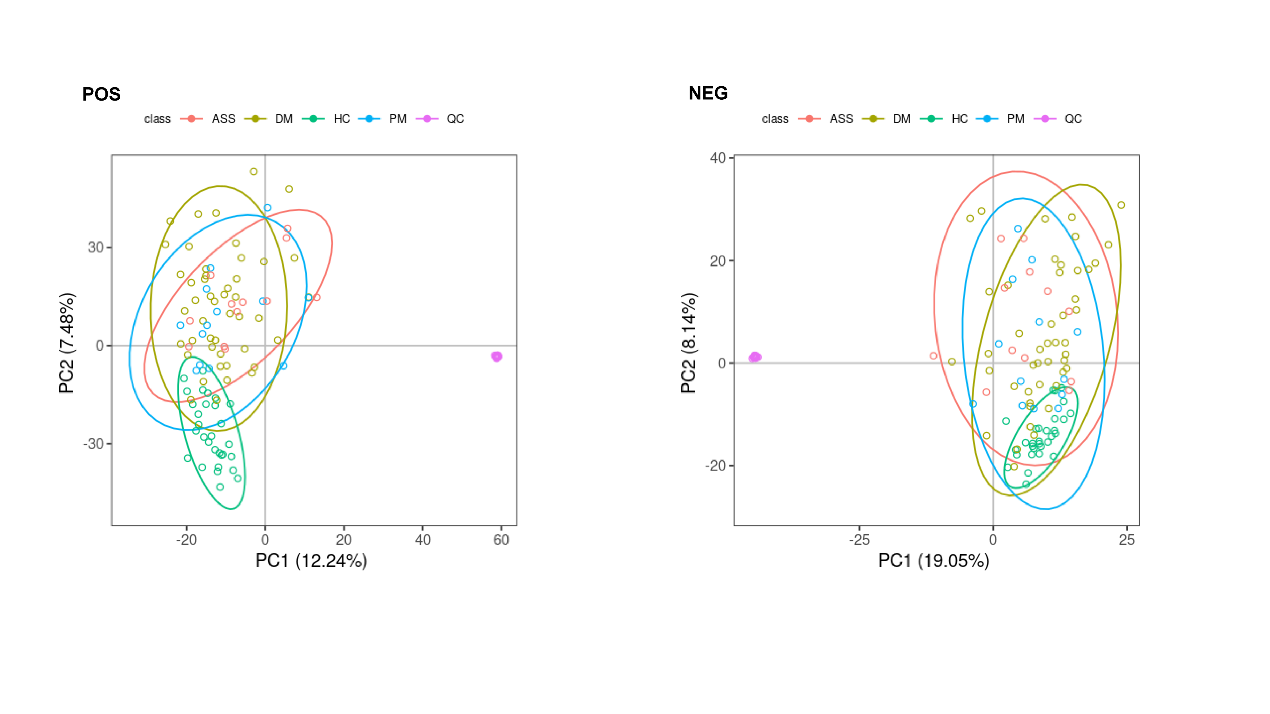


**Fig.S2 Principal component analysis (PCA) score plots with QC samples of plasma in the positive and negative modes.**

**POS**


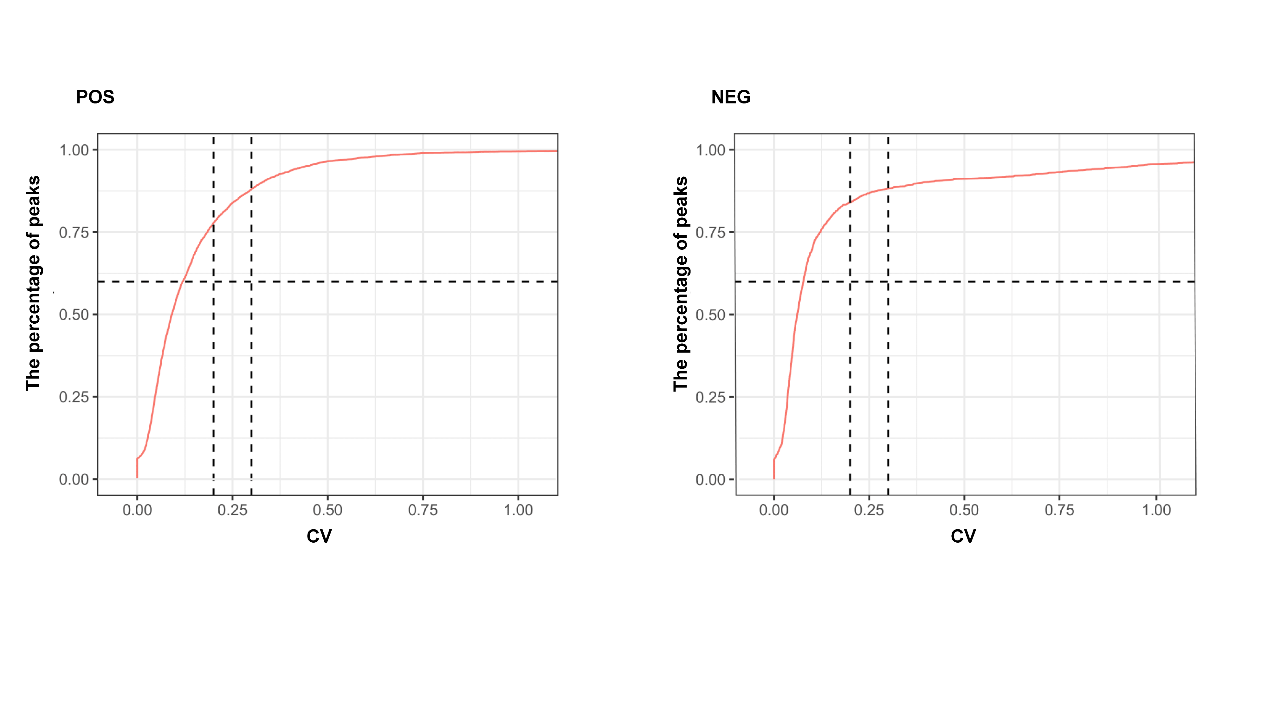


**NEG**

**Fig.S3 Coefficient of variation (CV) distribution of features in QC samples of plasma in the positive and negative modes.** The two lines perpendicular to the X axis are 20% and 30% CV reference line, respectively; and the line parallel to the X axis is 60% of the reference line.


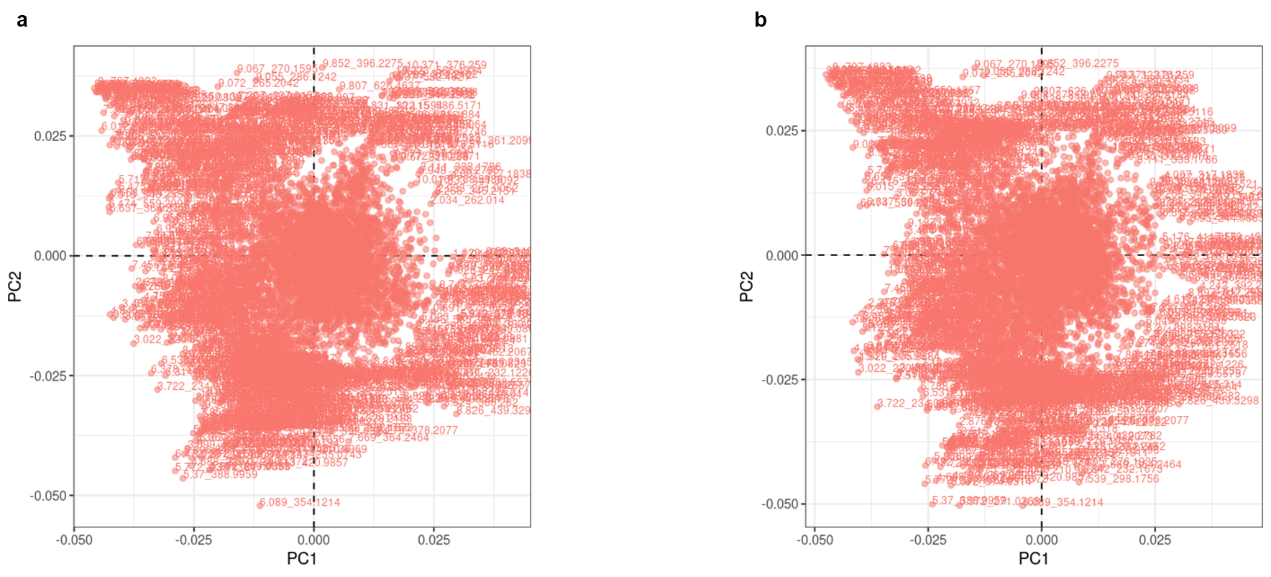


**Fig.S4 Principal component analysis (PCA) loading plots from all samples (a) and DM and HC (b) groups in plasma.**


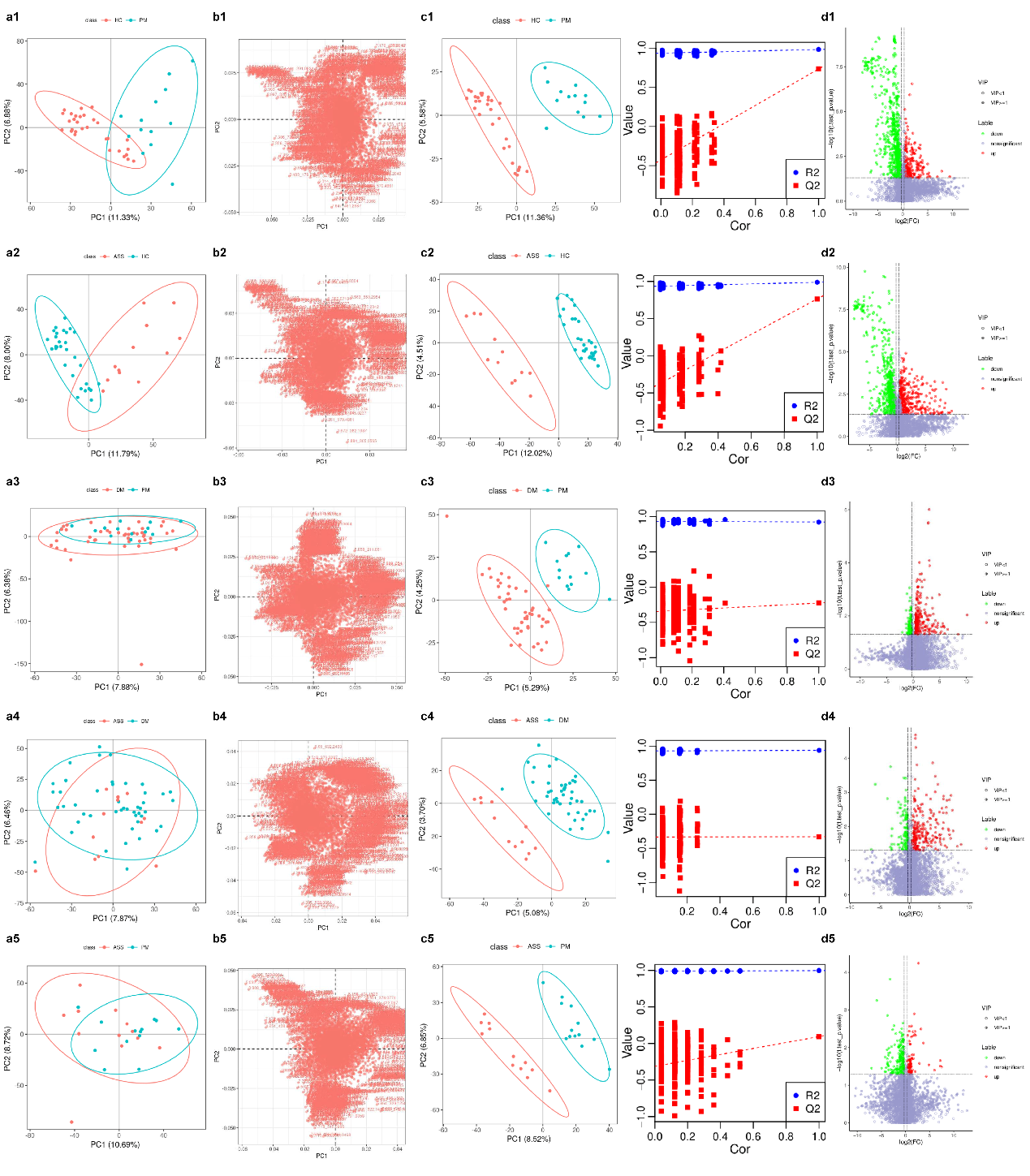


**Fig.S5 Principal component analysis (PCA) score plots, loading plots, partial least square discriminant analysis (PLS-DA) score plots, and Volcano plots from PM and HC (a1-d1), ASS and HC (a2-d2), DM and PM (a3-d3), DM and ASS (a4-d4), PM and ASS (a5-d5) groups in plasma.** PLS-DA models were evaluated by 200 permutation tests.


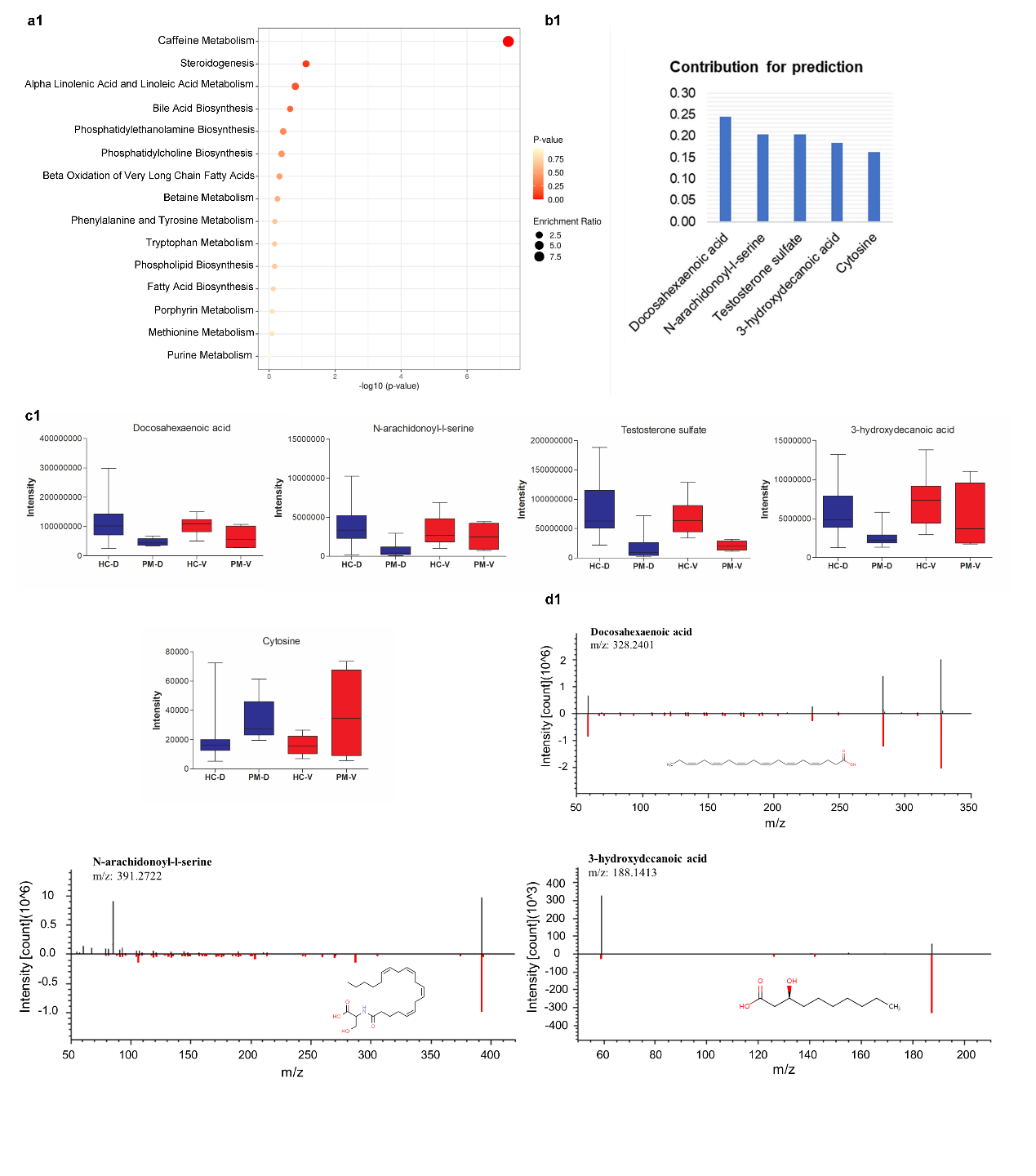


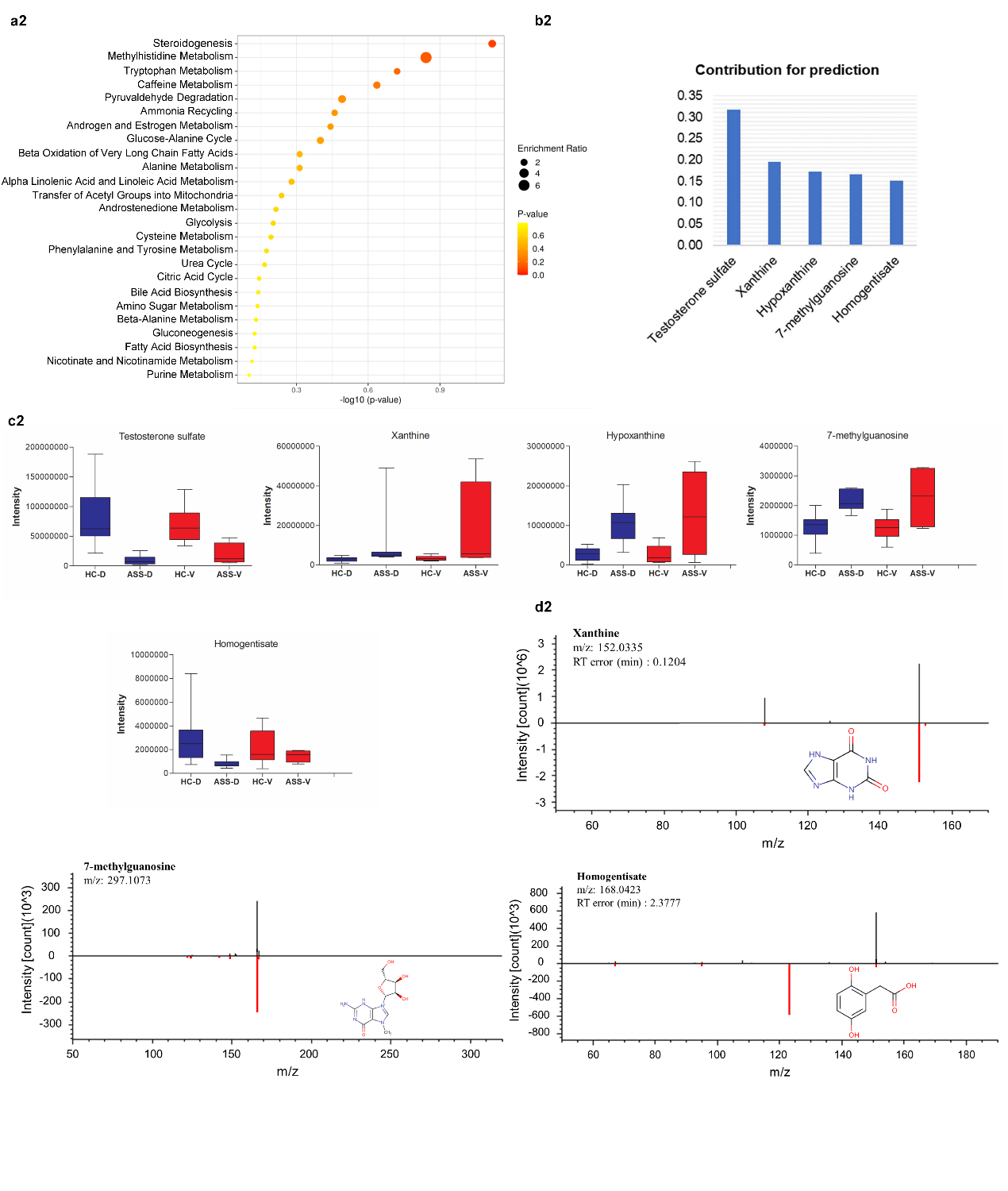
**Fig.S6 Five metabolites selected by random forest can identify polymyositis and anti-synthetase syndrome from healthy controls.** Enrichment analysis of differential metabolites with MSI level 1/2 between PM and HC (a1), ASS and HC (a2). Contribution of five metabolites to the identification model of PM (b1) and ASS (b2) from HC. The concentration trends of individual metabolites in the PM and HC (c1), ASS and HC (c2) groups in the discovery and validation sets. Detailed MS/MS spectra of Desoxycortone, N-arachidonoyl-l-serine, 3-hydroxydecanoic acid, Xanthine, 7-methylguanosine, and Homogentisate (d1, d2). HC-D, PM-D, HC and PM groups in the discovery set, respectively; HC-V, PM-V, HC and PM groups in the validation set, respectively; HC-D, ASS-D, HC and ASS groups in the discovery set, respectively; HC-V, ASS-V, HC and ASS groups in the validation set, respectively.


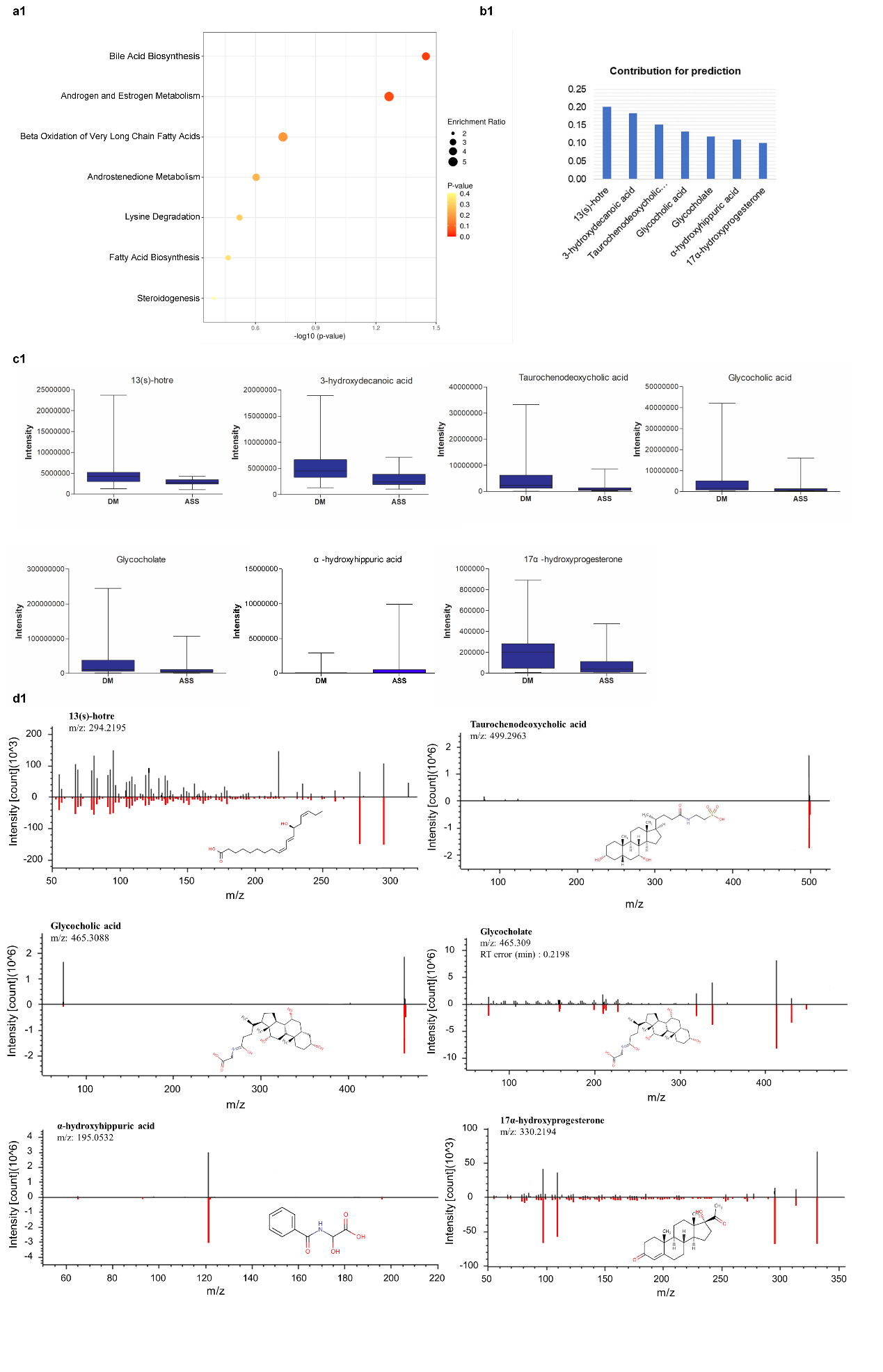


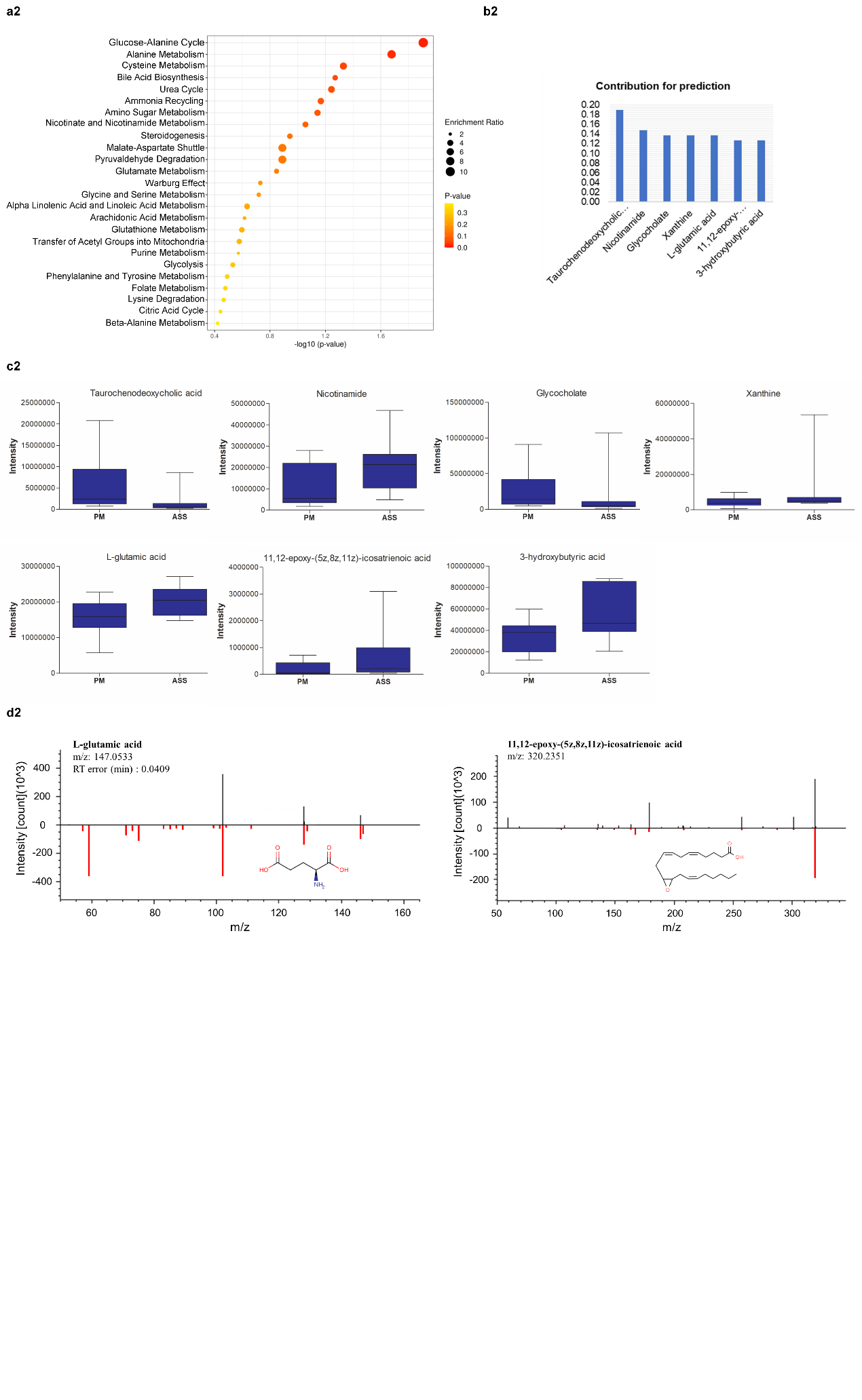


**Fig.S7 Five to seven metabolites selected by random forest can distinguish different idiopathic inflammatory myopathy subtypes.** Enrichment analysis of differential metabolites with MSI level 1/2 between DM and ASS groups (a1), PM and ASS groups (a2). Contribution of five, seven metabolites to the identification model of DM from ASS (b1), and PM from ASS (b2). The concentration trends of individual metabolites in the DM and ASS (c1), PM and ASS (c2) groups. Detailed MS/MS spectra of 13(s)-hotre, Taurochenodeoxycholic acid, Glycocholic acid, Glycocholate, α-hydroxyhippuric acid, 17α-hydroxyprogesterone, L-glutamic acid, and 11,12-epoxy-(5z,8z,11z)-icosatrienoic acid (d1, d2).


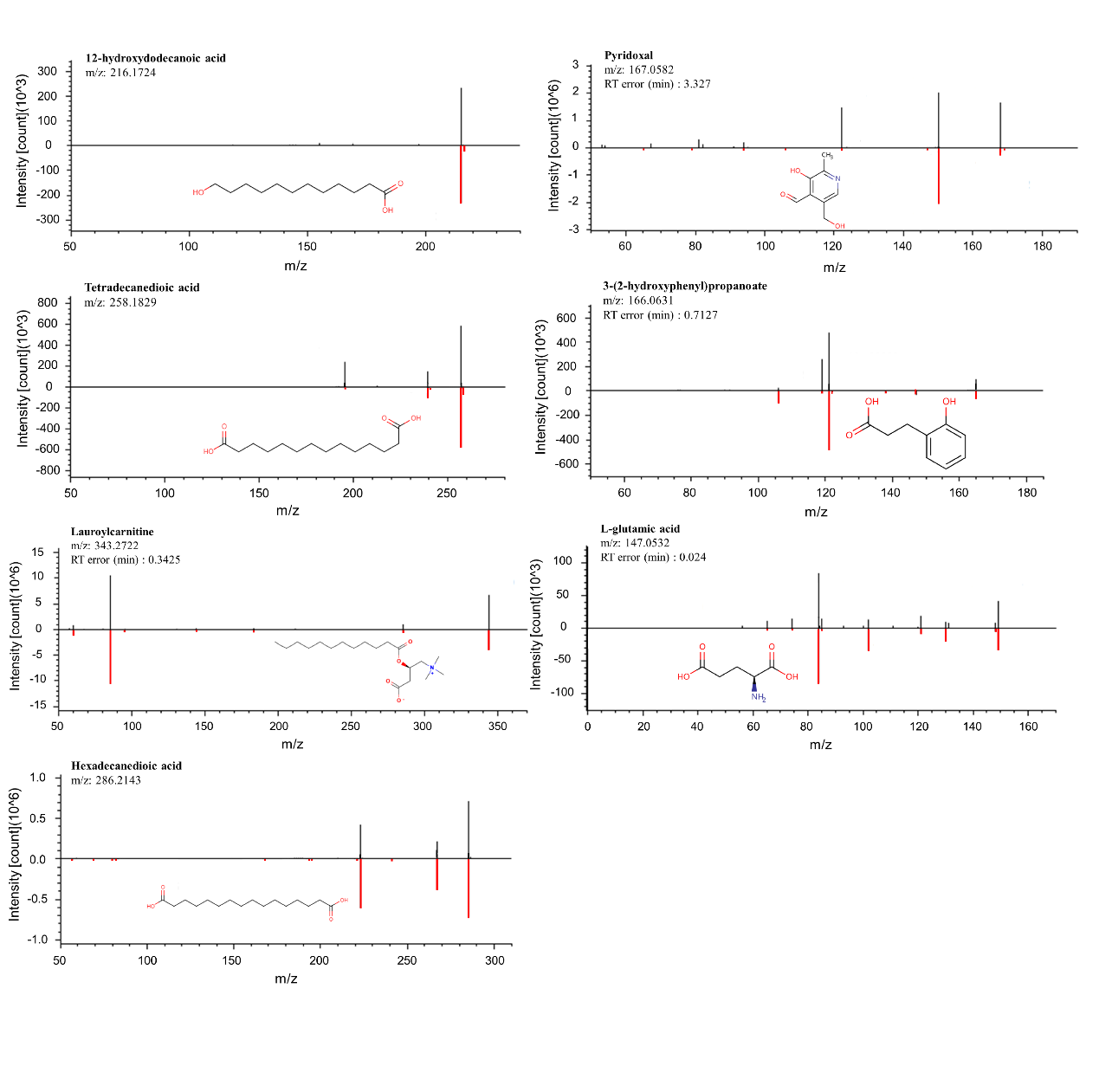


**Fig.S8 Detailed MS/MS spectra of seven potential metabolite biomarkers for prediction of MDA5+ DM.**

**Table S1. Sample list.**

| **Sample** | **Batch** | **Class** | **Order** |
| --- | --- | --- | --- |
| QC_06 | 1 | NA | 1 |
| QC_07 | 1 | NA | 2 |
| QC_08 | 1 | NA | 3 |
| QC_09 | 1 | NA | 4 |
| QC_10 | 1 | NA | 5 |
| HC-1 | 1 | HC | 6 |
| DM-6 | 1 | DM | 7 |
| HC-10 | 1 | HC | 8 |
| ASS-2 | 1 | ASS | 9 |
| DM-37 | 1 | DM | 10 |
| DM-15 | 1 | DM | 11 |
| ASS-9 | 1 | ASS | 12 |
| DM-16 | 1 | DM | 13 |
| ASS-8 | 1 | ASS | 14 |
| DM-23 | 1 | DM | 15 |
| QC_11 | 1 | NA | 16 |
| PM-8 | 1 | PM | 17 |
| HC-3 | 1 | HC | 18 |
| DM-29 | 1 | DM | 19 |
| HC-21 | 1 | HC | 20 |
| ASS-10 | 1 | ASS | 21 |
| PM-4 | 1 | PM | 22 |
| DM-19 | 1 | DM | 23 |
| HC-4 | 1 | HC | 24 |
| DM-21 | 1 | DM | 25 |
| QC_12 | 1 | NA | 26 |
| HC-6 | 1 | HC | 27 |
| HC-28 | 1 | HC | 28 |
| PM-11 | 1 | PM | 29 |
| HC-8 | 1 | HC | 30 |
| DM-40 | 1 | DM | 31 |
| HC-30 | 1 | HC | 32 |
| HC-7 | 1 | HC | 33 |
| ASS-5 | 1 | ASS | 34 |
| DM-27 | 1 | DM | 35 |
| QC_13 | 1 | NA | 36 |
| HC-11 | 1 | HC | 37 |
| DM-26 | 1 | DM | 38 |
| DM-32 | 1 | DM | 39 |
| DM-22 | 1 | DM | 40 |
| ASS-7 | 1 | ASS | 41 |
| HC-12 | 1 | HC | 42 |
| PM-6 | 1 | PM | 43 |
| DM-12 | 1 | DM | 44 |
| HC-17 | 1 | HC | 45 |
| QC_14 | 1 | NA | 46 |
| ASS-12 | 1 | ASS | 47 |
| DM-35 | 1 | DM | 48 |
| HC-22 | 1 | HC | 49 |
| HC-15 | 1 | HC | 50 |
| PM-2 | 1 | PM | 51 |
| DM-18 | 1 | DM | 52 |
| HC-13 | 1 | HC | 53 |
| HC-19 | 1 | HC | 54 |
| PM-12 | 1 | PM | 55 |
| QC_15 | 1 | NA | 56 |
| DM-43 | 1 | DM | 57 |
| HC-27 | 1 | HC | 58 |
| HC-5 | 1 | HC | 59 |
| PM-1 | 1 | PM | 60 |
| DM-39 | 1 | DM | 61 |
| HC-24 | 1 | HC | 62 |
| ASS-3 | 1 | ASS | 63 |
| DM-25 | 1 | DM | 64 |
| HC-18 | 1 | HC | 65 |
| QC_16 | 1 | NA | 66 |
| HC-25 | 1 | HC | 67 |
| ASS-6 | 1 | ASS | 68 |
| DM-24 | 1 | DM | 69 |
| DM-14 | 1 | DM | 70 |
| HC-23 | 1 | HC | 71 |
| DM-1 | 1 | DM | 72 |
| DM-45 | 1 | DM | 73 |
| HC-29 | 1 | HC | 74 |
| HC-9 | 1 | HC | 75 |
| QC_17 | 1 | NA | 76 |
| DM-31 | 1 | DM | 77 |
| HC-26 | 1 | HC | 78 |
| DM-4 | 1 | DM | 79 |
| ASS-11 | 1 | ASS | 80 |
| DM-36 | 1 | DM | 81 |
| DM-17 | 1 | DM | 82 |
| PM-9 | 1 | PM | 83 |
| DM-8 | 1 | DM | 84 |
| PM-5 | 1 | PM | 85 |
| QC_18 | 1 | NA | 86 |
| DM-13 | 1 | DM | 87 |
| HC-20 | 1 | HC | 88 |
| DM-41 | 1 | DM | 89 |
| DM-11 | 1 | DM | 90 |
| DM-20 | 1 | DM | 91 |
| HC-2 | 1 | HC | 92 |
| PM-3 | 1 | PM | 93 |
| DM-2 | 1 | DM | 94 |
| ASS-4 | 1 | ASS | 95 |
| QC_19 | 1 | NA | 96 |
| DM-33 | 1 | DM | 97 |
| PM-13 | 1 | PM | 98 |
| DM-9 | 1 | DM | 99 |
| DM-5 | 1 | DM | 100 |
| DM-46 | 1 | DM | 101 |
| DM-10 | 1 | DM | 102 |
| DM-30 | 1 | DM | 103 |
| HC-16 | 1 | HC | 104 |
| DM-7 | 1 | DM | 105 |
| QC_20 | 1 | NA | 106 |
| DM-3 | 1 | DM | 107 |
| DM-28 | 1 | DM | 108 |
| DM-34 | 1 | DM | 109 |
| DM-38 | 1 | DM | 110 |
| PM-7 | 1 | PM | 111 |
| HC-14 | 1 | HC | 112 |
| DM-42 | 1 | DM | 113 |
| PM-10 | 1 | PM | 114 |
| DM-44 | 1 | DM | 115 |
| QC_21 | 1 | NA | 116 |
| ASS-1 | 1 | ASS | 117 |
| QC_22 | 1 | NA | 118 |
| QC_23 | 1 | NA | 119 |
| QC_24 | 1 | NA | 120 |

**Table S2. P values for comparing IIM subtype and HC groups in the discovery and validation cohorts.**

| **Group** | **Characteristic** | ***P* value** | |
| --- | --- | --- | --- |
|  |  | Discovery cohort | Validation cohort |
| **DM** vs **HC** | Age^1^ | 0.907 | 0.27 |
|  | Gender^2^ | 0.927 | 1 |
| **PM** vs **HC** | Age^1^ | 0.704 | 0.512 |
|  | Gender^2^ | 0.12 | 0.597 |
| **ASS** vs **HC** | Age^1^ | 0.205 | 0.628 |
|  | Gender^2^ | 0.159 | 0.597 |
| **DM** vs **PM** | Age^1^ | 0.718 | - |
|  | Gender^2^ | 0.11 | - |
| **DM** vs **ASS** | Age^1^ | 0.614 | - |
|  | Gender^2^ | 0.145 | - |
| **PM** vs **ASS** | Age^1^ | 0.471 | - |
|  | Gender^2^ | 0.93 | - |
| **MDA5+ DM** vs **MDA5- DM** | Age^1^ | 0.33 | 0.108 |
|  | Gender^2^ | 0.788 | 0.264 |

^1^Differences in age between the different groups were tested using the two-tailed unpaired t test. ^2^Differences in gender between the different groups were tested using the Fisher exact test. HC, healthy control; IIM, idiopathic inflammatory myopathy.
